# Controllable synthesis of Pd and Pt shells on Au nanoparticles with electrodeposition

**DOI:** 10.1038/s41598-024-84476-z

**Published:** 2025-01-08

**Authors:** Mohsen Elabbadi, Christina Boukouvala, Emilie Ringe

**Affiliations:** 1 Department of Materials Science and Metallurgy, University of Cambridge, 27 Charles Babbage Road, Cambridge, CB3 0FS UK; 2 Department of Earth Sciences, University of Cambridge, Downing Street, Cambridge, CB2 3EQ UK

**Keywords:** Bimetallic nanoparticles, Electrodeposition, Nanoplasmonics, Spectroelectrochemistry, Localized surface plasmon resonance, Nanoparticles, Nanoparticles

## Abstract

Shells of Pd and Pt were synthesized on Au nanoparticles by electrodeposition, leading to controllable size and optical properties. This approach yielded core–shell structures with good homogeneity in size after the optimization of electrochemical parameters such as deposition current and charge transfer, as well as nanoparticle surface treatment. Dark field scattering microscopy and spectroscopy were used to track changes in the optical response of individual particles during deposition. The deposition of thin shells of Pd or Pt initially leads to a damping of the Au localized surface plasmon resonance which is followed by its redshift and an increase in scattering intensity. These changes were rationalized with numerical calculations and correlated with electron microscopy analyses revealing the morphology and thickness of Pd and Pt shells. This electrochemical approach provides a new pathway for the synthesis of bimetallic structures with catalytic surfaces.

## Introduction

In bimetallic nanoparticles (NPs), the ability to control the core and shell composition and distribution enables multi-functionality, where the core and shell can provide bulk or surface properties including magnetic, optical, catalytic, and so on. The combination of a metallic core capable of localized surface plasmon resonances (LSPRs), coherent oscillations of conduction electrons, and a catalytic surface has attracted significant attention in the last decade, owing to the resulting phenomenon of plasmon-enhanced catalysis^1–3^.

Core–shell or decorated NPs are commonly synthesized by colloidal reduction, where one metal is first reduced to form the core, followed by the subsequent reduction of a second metal to form a shell or particles on its surface. A gradient composition can also be obtained through co-reduction when metals have different reduction potentials^4^. Alternatively, bimetallics can be produced by the partial galvanic replacement of a metal core such as Ag, Cu, Al, or Mg by a more noble metal such as Au, Pd, or Pt^5–10^.

Electrodeposition is a less commonly studied approach to create bimetallic nanostructures, including those combining plasmonic and catalytic properties. Compared to galvanic replacement where the reduction potential difference between the two metals provides the driving force for electron transfer, in electrodeposition the potential is directly and arbitrarily externally controlled. This approach provides the opportunity to select and modify the electron transfer rate (current) and the total number of electrons transferred (total charge transfer), thus controlling the number of metal ions reduced.

An early study by Novo et al*.* showed that applying an electrical potential to Au NPs resulted in a change in the LSPR frequency attributed to a change in electron density^11^; Byers et al*.* later demonstrated the heterogeneity of these changes^12^. Subsequent reports using spectroelectrochemical techniques have investigated various related phenomena such as the electrodeposition and oxidation of single Ag NPs^13–15^, the photooxidation of Ag^16^, the oxidation of Ag NPs in the presence of Cl^-^^17^, as well as the electrochemical amalgamation of Hg on Au NPs^18,19^.

While these spectroelectrochemical techniques have revealed much about non-faradaic and oxidation / reduction processes in metallic NPs, little work to date has investigated the use of electrodeposition in the synthesis of plasmonic bimetallic NPs. Chirea et al*.* first electrodeposited Ag onto Au nanostars and correlated in situ dark field optical spectroscopy with scanning electron microscopy (SEM) to study the effect of composition changes on the plasmonic behavior^20^. Ag deposition on nanostars resulted in a blue shift, and some investigation of electrochemical parameters was performed. Oh et al*.* used electrodeposition to form multi-lobed Cu on Au nanocubes, tracking changes in their optical properties by converting scattering color to a peak wavelength^21^. They produced three distinct morphologies by systematically varying the deposition sweep rate. Although they were unable to directly observe it, they hypothesized that underpotential deposition was a critical step in controlling nanoscale morphology. Later, Hu et al. observed both bulk electrodeposition and underpotential deposition of Ag on Au octahedra and cubes by using a highly sensitive dark field spectroscopy setup based on a dual immersion objective and condenser lens system^22^. Going beyond purely metallic NP systems, Wang et al*.* monitored in situ the electrodeposition of semiconductor (CdS, CdSe, and ZnS) shells on Au spheres with dark field microscopy, demonstrating control of shell thickness with increasing deposition time^23^. We recently reported^24^ the electrodeposition of catalytically relevant^25^ Cu on Au NPs, achieving good homogeneity through controlled current electrodeposition, and monitored the optical response of single NPs by optical dark field spectroscopy correlated with electron microscopy.

A metallic combination of particular interest, due to its oxidative stability and combination of plasmonic and catalytic properties is Au with noble metal catalysts Pt and Pd. Wang et al*.* demonstrated an enhanced rate and yield of the Suzuki coupling reaction from illumination alone using plasmonic Pd on Au rod nanostructures^26^, while Pt on Au nanorods was efficient in the photocatalytic evolution of H_2_^27^. The plasmonic response and electric field distributions are sensitive to the shape and location of the Pd or Pt decorations; accordingly this morphology dependence is translated across to the photocatalytic performance^28,29^. Simultaneous control of nanoparticle morphology and optical properties is therefore key to designing efficient photocatalytic systems.

While the electrodeposition of Pd and Pt on substrates such as Au is well established^30,31^, only fairly recently have these metals been deposited on nanostructures of Au, typically for electrocatalysis applications^32,33^. Xu et al*.* used dark field microscopy to track the electrodeposition of shells of Pt, Pd, Rh, PtPd and PdRh onto 50 nm Au NPs, investigating these plasmonic systems for the photo and electrocatalytic oxidation of methanol^34^. Their study highlighted the potential for such structures in catalysis, and opened the door for a more in-depth understanding of the factors controlling shell thickness and its implication for the optical properties of the resulting structures.

Here we show control of the extent of Pd and Pt electrodeposition on Au NPs, demonstrating fine tuning of the shell thickness in these bimetallic nanostructures. A combined hyperspectral spectroelectrochemical approach was used to track the plasmonic response of single NPs during deposition, revealing the rapid nucleation of the shell metal. Scattering intensity and spectral changes in the plasmonic response after deposition provided details of the shell effect on the plasmonic behavior, which were supported by numerical results obtained in the discrete dipole approximation (DDA). The experimental parameters, such as deposition current, charge transfer, and NP surface treatment, were optimized to produce uniform bimetallic Au core- Pt and Pd shell NPs with good size and morphology homogeneity. This study demonstrates that electrodeposition can be used for the synthesis of bimetallic plasmonic-catalytic particles with tunable composition and plasmonic properties.

## Methods

### Au NP synthesis

**Fig. 1:**
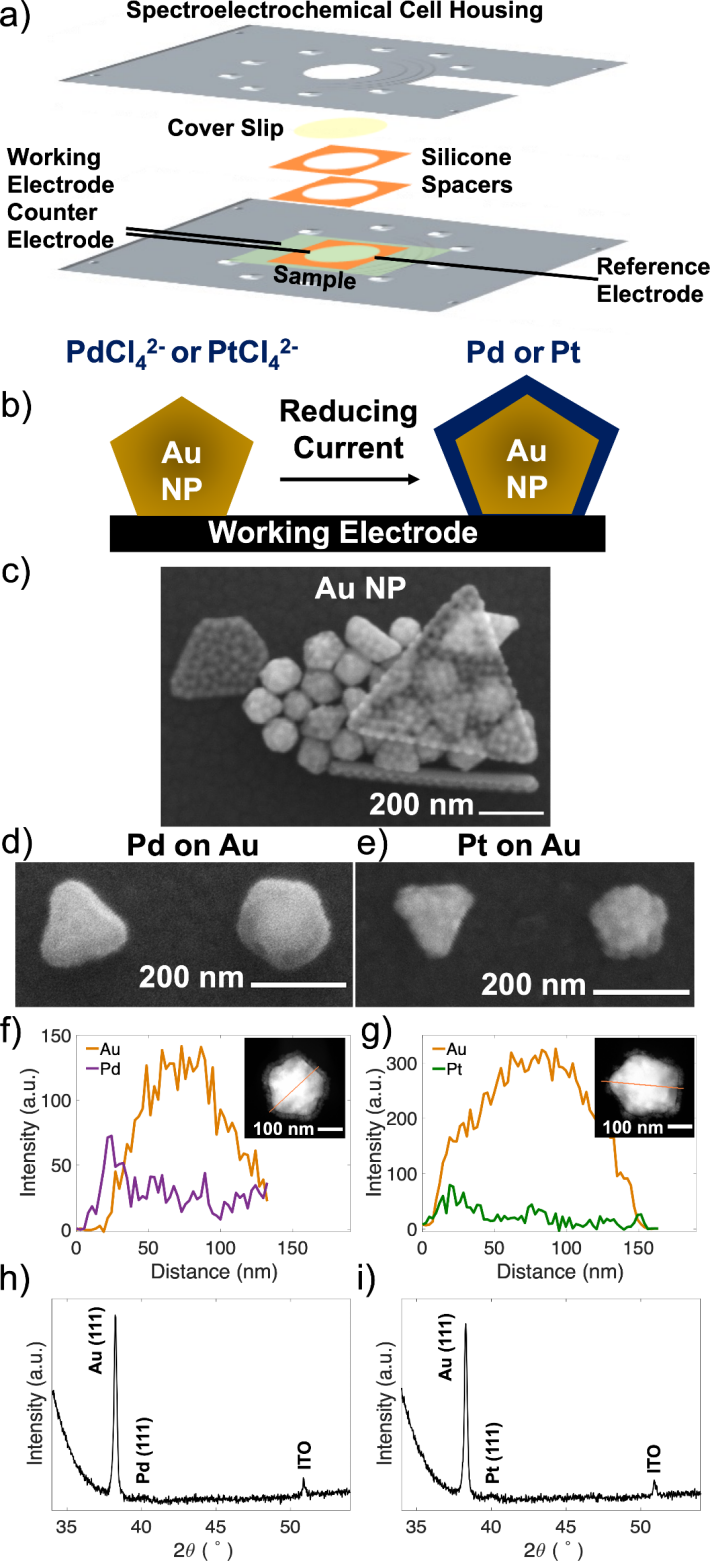
Overview of Pd and Pt electrodeposition on Au NPs. **a**) Spectroelectrochemical cell schematic, **b**) Pd or Pt electrodeposition on a single Au decahedral NP, **c**) SEM image of plasma treated Au NPs deposited at high density to show the mixture of shapes including decahedra, isocahedra, and truncated bitetrahedra, SEM images of Au NPs after **d**) Pd and **e**) Pt electrodeposition, STEM-EDS line scan of **f**) Pd and **g**) Pt on Au NPs, where the scan follows the orange line on the STEM-HAADF image (inset), and XRD pattern of **h**) Pd and **i**) Pt on Au NPs with the substrate (ITO) peak labeled.

Tetrachloroaurate trihydrate (HAuCl_4_·3H_2_O, 99.9 + %), poly(vinylpyrrolidone) (PVP, M_W_ 55,000), and diethylene glycol (DEG, 99%) were purchased from Sigma-Aldrich and used without further purification. Au NPs were synthesized following by Seo et al.’s published procedure^35^, resulting in a mixture of mostly decahedra, icosahedra, and truncated bitetrahedra (Fig. 1). 3.5 g of PVP was dissolved in 12.5 mL of DEG and refluxed for 5 min. A solution of 10 mg of HAuCl_4_·3H_2_O in 1 mL of DEG was then added to the reaction mixture and refluxed for a further 10 min. The mixture was diluted with 12.5 mL of ethanol after cooling to room temperature, followed by centrifugation at 6000 rpm for 30 min. Ethanol addition and centrifugation was repeated 4 times.

### Spectroelectrochemistry

Electrodeposition was performed and tracked using a three electrode spectroelectrochemical cell. Indium tin oxide (ITO) coated slides (SPI supplies, 8–12 Ω resistance) were cleaned by immersion into a 1:1:5 mixture of NH_4_OH (35 wt % solution in water):H_2_O_2_ (30 wt % solution in water):H_2_O at 70 °C for 1 min, and then rinsed with deionized water. Au NPs were drop cast onto the slides to form the working electrode. A Pt mesh (Alfa Aesar, 100 mesh woven, 0.0762 mm diameter wire, 99.9% purity) was used as the counter electrode, while the reference electrode was a 1 mm barrel diameter Ag/AgCl electrode (Alvatek). A custom designed cell suitable for an optical microscope stage held all three electrodes in place and contained the reaction solution (Fig. 1a). Silicone isolators (Grace Bio-Laboratories, 13 mm diameter opening) acted as spacers in the cell to separate the electrodes and ensure a leak-proof seal, leading to a cell area of 1.3 cm^2^. The aqueous electrolyte solution (approximately 270 μL) contained 0.50 mM Na_2_PdCl_4_ (99.8% from Sigma-Aldrich) or 1.0 mM Na_2_PtCl_4_.H_2_O (99.95% from Alfa Aesar) except for concentration studies where they were varied from 0.5 mM to 2.0 mM. A supporting electrolyte, H_2_SO_4_ (Sigma-Aldrich, 99.9%), was present in 1 mM concentration. The electrochemical experiments were controlled by a Gamry Reference 600 potentiostat.

### Dark field optical microscopy

Single particle scattering data were obtained with dark field optical microscopy as described in ref.^36^ A 12 V 100 W halogen lamp (Nikon D-LH/LC) illuminated the sample through a 0.95–0.80 numerical aperture (NA) dark field condenser (Nikon), and scattered light was collected by a 0.5 NA objective (Nikon CFI Plan Fluor 100XS oil) through an inverted optical microscope (Nikon Eclipse Ti2).

For spectral measurements, dispersion by a Princeton Instruments IsoPlane SCT320 spectrometer equipped with a 50 g/mm grating was followed by detection with a Princeton Instruments ProEM HS 1024 × 1024 EMCCD. Dark field optical scattering images were obtained with a Thorlabs CS505CU—Kiralux 5.0 MP Color CMOS Camera. The stage position was controlled by a Physik Instrumente P-545.3C7 piezoelectric stage.

Spectral time series data were acquired at 0.25 s intervals with the NPs in solution. Hyperspectral measurements, following the approach described in ref.^36^ were acquired ex situ before and after deposition, following rinsing with deionized water and drying, with a step size of 0.3 μm and an acquisition time of 2 s per step.

### Electron microscopy and X-ray diffraction

SEM was performed using a Quanta-650F FEG-SEM operated at 15 kV using Everhart–Thornley (ET) and concentric backscattered (CBS) electron detectors. Samples for high-angle annular dark field scanning transmission electron microscopy (HAADF-STEM) were prepared by sonicating the ITO-coated slide in isopropanol to obtain dispersed NPs, which were subsequently drop cast on Cu-C grids. HAADF-STEM was performed on a FEI Tecnai Osiris operated at 200 kV; STEM-energy dispersive X-ray spectroscopy (EDS) was performed using a Bruker Super-X quad detector. A Bruker D8 DAVINCI with position sensitive detector and Cu Kα radiation was used to produce X-ray diffraction (XRD) data.

### Data processing

A custom MATLAB graphical user interface was used to analyze the optical data acquired from hyperspectral microscopy and extract single particle spectra. The stacking together of *y x*–λ data files created a 3D *x*–*y*–λ datacube, while for time-resolved data, a 3D *x*–*t*–λ datacube was obtained by stacking together *t x*–λ data files. The raw data were corrected for lamp profile, dark counts and background as described in ref.^36^.

Individual NPs were identified and localized by manually setting a threshold creating an intensity isoline which defines the NP scattering border and its center. Intensities were then integrated over a 3 × 4 pixels region which corresponds to a 0.90 × 0.56 μm area. 120–130 NPs were selected per sample.

For the color camera data, color thresholding was used in ImageJ to automatically select particles with an area range of 50 – 1000 pixels. The intensity from each of the RGB color channels was summed to give the intensity at each pixel, with the maximum intensity within that particle area taken as the scattering intensity of the particle. This was performed for time-resolved studies with frames acquired at 1 s intervals.

### Numerical simulations

Scattering cross sections and field distributions were obtained by numerical calculation through solving Maxwell’s equations in the discrete dipole approximation using DDSCAT^37^ for the decahedral shapes and via a transfer-matrix method using STRATIFY^38^ for spheres. The frequency dependent refractive index of Au was taken from Johnson and Christy^39^, while those of Pd and Pt were from Werner^40^. The ambient refractive index was set to 1.3 to approximate the ITO substrate and air^41^.

The unpolarized light and the light cone generated by the dark field condenser of the experimental setup were approximated by an orthogonally polarized field incident at an angle of 31° to the substrate. The core NP was modelled as a decahedron of tip to edge length 130 nm (longest tip to tip length 136.6 nm, effective radius of 45.7 nm) and an interdipole distance of 2 nm was used in all calculations. The electric field output was visualized using the Paraview software.

## Results and discussion

### Controllable Pd and Pt deposition on Au

Pd and Pt were electrodeposited on Au NPs in a transparent liquid cell (Fig. 1a). Au NPs were synthesized following the procedure by Seo et al.^35^, forming a mixture of PVP-capped decahedra, isocahedra, and truncated bitetrahedra with average sizes of 129 ± 6 nm (longest tip to tip length), 119 ± 6 nm (tip to tip), and 133 ± 7 nm (tip to edge), respectively. Additional shapes such as rods or large triangular shapes were occasionally seen in the synthesis mixture, as included in Fig. 1. These Au NPs were treated with O_2_/Ar plasma with a Quorum K1050X for 5 min at 20 W to remove PVP, leading to roughening of their surface (Fig. 1c and additional discussion in the Supporting Information) with triangular pitting as reported elsewhere^42^. The NPs were drop cast on working electrode ITO-coated glass coverslips, and the cell was filled with aqueous Na_2_PdCl_4_ or Na_2_PtCl_4_ in a background electrolyte of H_2_SO_4_ then sealed. After deposition optimization spanning multiple parameters as discussed in the SI and Supplementary Fig. S1-S9, we used controlled current deposition with typical depositions of 10 µA (5 s) for Pd and 50 µA (10 s) for Pt (Fig. 1d-e).

Pd and Pt deposited selectively on the surface of Au NPs. Deposition occurred as a conjoined lumpy shell for both metals, with multiple nucleation sites occurring across each Au NP, as evidenced by SEM and STEM HAADF images (Fig. 1 and Supplementary Fig. S10). SEM images further reveal that at lower charge transfers, deposition of Pd and Pt is selective on Au NPs, *i.e.*, with no deposition seen on the ITO substrate.

The Pd or Pt surface composition and their distribution as a shell around Au were confirmed by STEM energy dispersive X-ray spectroscopy (STEM-EDS) (Fig. 1f-g and Supplementary Fig. S10-11). This composition is supported by powder X-ray diffraction (XRD) data, in which broad peaks with low diffraction intensity suggest the presence of nanosized grains of Pd and Pt (Fig. 1h-i).

**Fig. 2:**
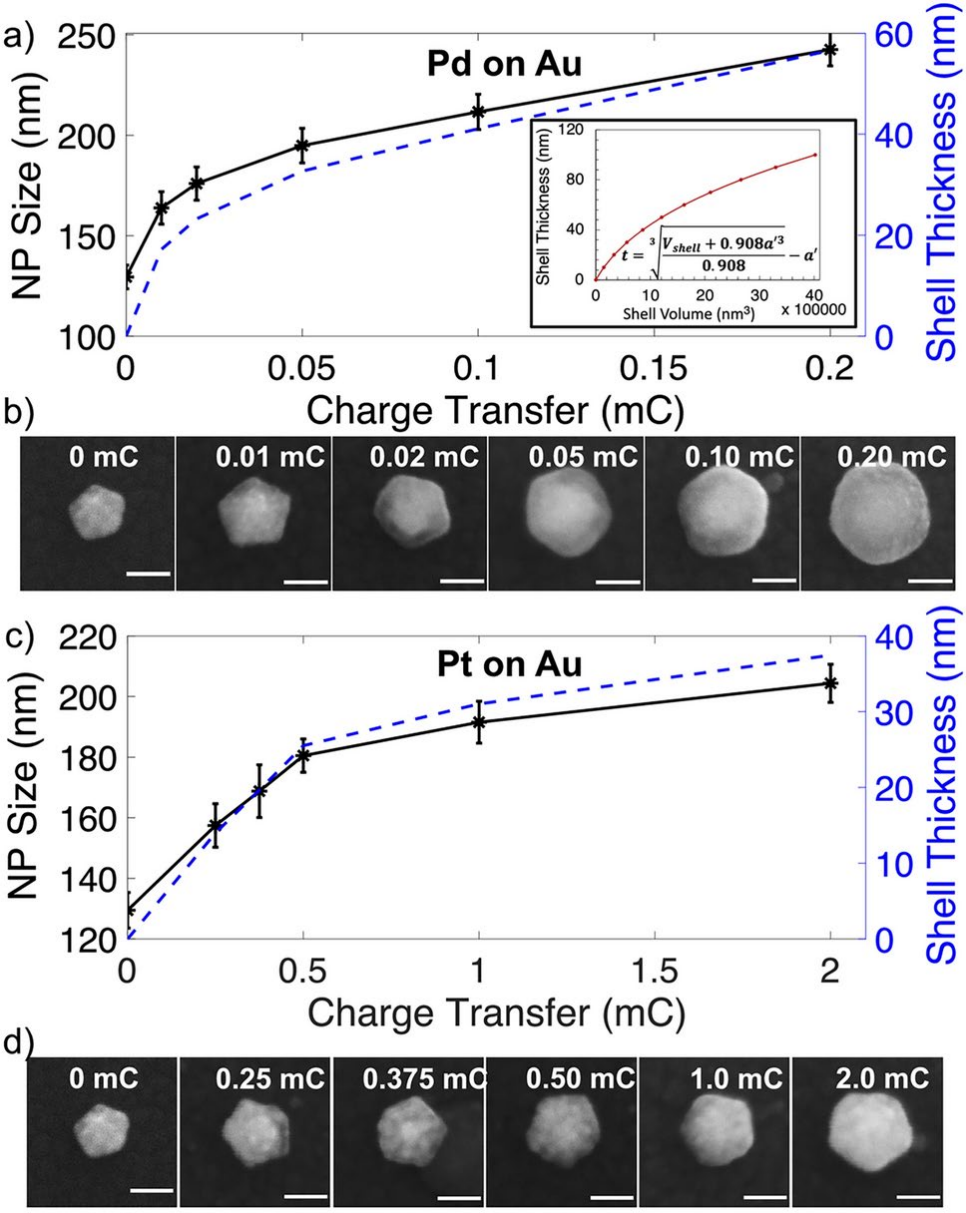
Control of NP size with charge transfer. NP size (tip to tip length of decahedra, starting size of 129 nm) and associated shell thickness against charge transfer of **a**) Pd and **c**) Pt on Au NPs, SEM images of representative **b**) Pd and **d**) Pt on Au NPs. All scale bars, 100 nm; the same error applies to the size and shell thickness and was omitted on the thickness for clarity. A minimum of 30 NPs were measured for each data point. The inset in **a**) shows the analytical trend of a shell of constant volume increase as derived in Schematic S1.

Control of the Pd or Pt shell thickness was achieved by varying the total charge transfer (atoms reduced) by varying deposition time with a constant current. Charge transfer spanned the range 0.01 to 0.2 mC for Pd and 0.25 to 2.0 mC for Pt at constant currents of 10 µA and 50 µA, respectively. Figure 2a and c show the measured NP sizes of decahedra, the majority product of the synthesis, while 2b and 2d show representative SEM images; further images of these depositions are shown in Fig. 4. These results demonstrate good thickness control with narrow spreads, with up to 57 nm shells for Pd and 37 nm shells for Pt. The initial growth rate of the shell is fast then decreases with longer times, with early shells being lumpy structures that become smoother and more filled in as they grow and deposition proceeds.

The growth pattern can be compared to an analytical model. The inset of Fig. 2a (derived and described in the SI) shows the expected shell growth on a decahedron assuming deposition at a constant volume rate as expected from a constant current experiment. Because of the overall growth of the NP, the shell thickness growth rate decreases with increasing total shell volume. The experimental data appear to plateau more quickly than the model, indicating a change in volume deposition rate on the NP during the experiment. We hypothesize that deposition of Pd or Pt elsewhere than on the growing NPs^43^ (plausibly due to depletion of available ions immediately surrounding them) is responsible for this effect, consistent with the observation of deposition on the ITO slide at high charge transfer.

### Effect of deposition on the plasmonic response

Optical microscopy was used to quantify and track the extent of metal deposition. NP scattering spectra and color camera images were obtained before and after deposition using a dark field optical setup and a transparent conductive oxide support (ITO) (Fig. 1a, 3a). To maximize the scattering signal, measurements were performed in air; post-deposition samples were rinsed, dried, and then measured. Scattering spectra were correlated to post-deposition morphologies obtained with SEM.

**Fig. 3:**
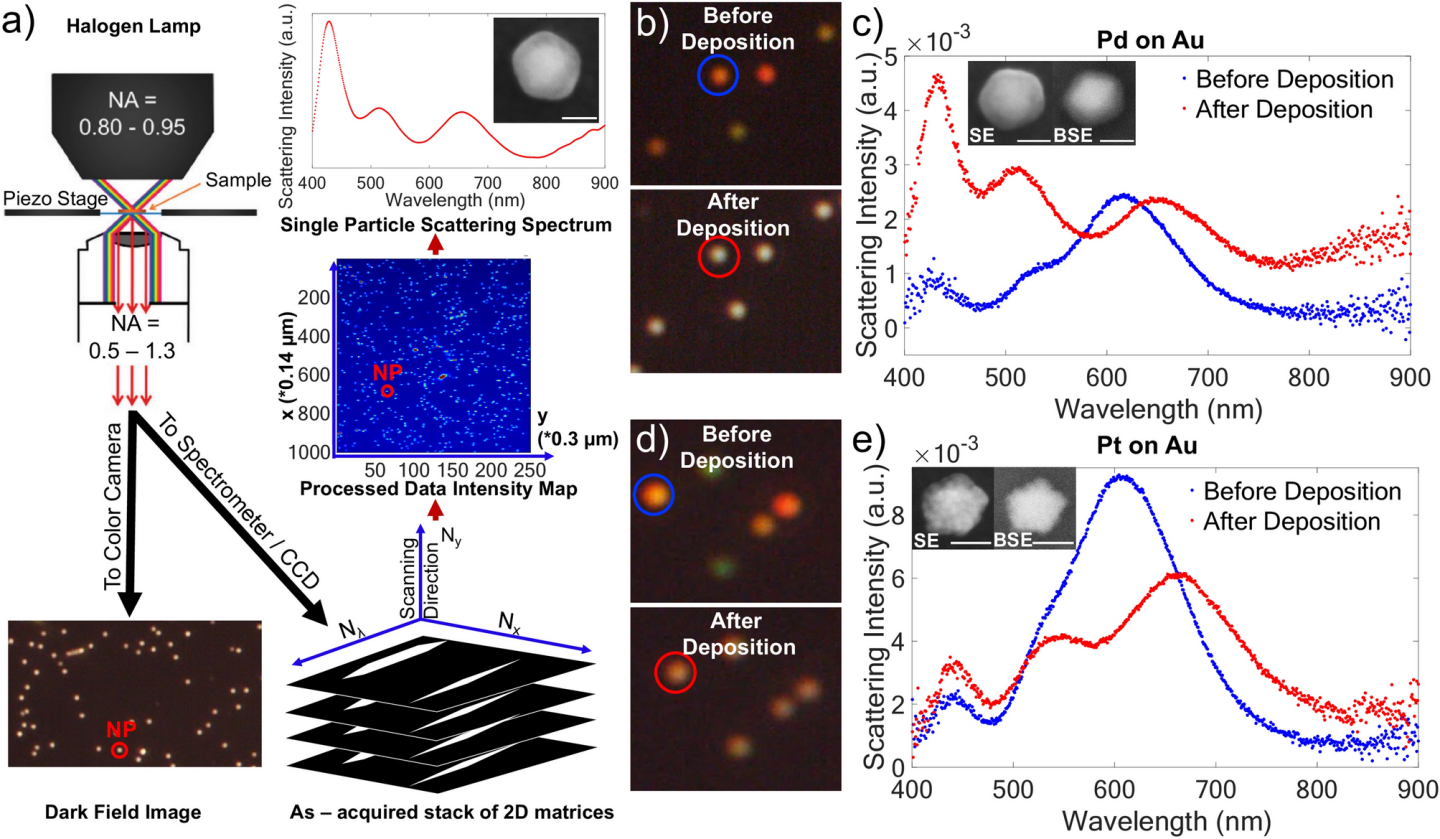
Optical characterization of the electrodeposition of Pd or Pt on Au NPs. **a**) Schematic of the setup used for dark field microscopy, showing dark field color camera imaging, hyperspectral data acquisition and processing, and SEM-spectrum correlation, **b**) dark field color images before and after Pd deposition (5 s, 10 µA) with NP in **c**) circled, and **d**) Pt deposition (10 s, 50 µA) with NP in **e**) circled, scattering spectrum of NP before and after deposition, with SE and BSE images of that NP for **c**) Pd and **e**) Pt deposition. Scale bars, 100 nm. Dark field microscopy schematic in (**a**) reproduced from ref.^44^ with permission. Copyright 2013 Royal Society of Chemistry.

The plasmonic response of Au NPs is affected by the addition of a Pd or Pt shell, as can be seen by optical approaches. Simple color camera images reveal a color change after deposition; Fig. 3b and d provide examples for Pd (5 s, 10 µA) and Pt (10 s, 50 µA). A color change from orange or green (arising from the different shapes of Au NPs) to nearly white was observed for Pd, while a slight reddening and dimming was observed for Pt. Corresponding scattering spectra are shown in Fig. 3c and e. The increase in blue scattering explains the shift to white color observed for Pd, while, for Pt, the redshift of the low energy peak and the overall decrease in intensity matches well with the dimming observed in the color camera. Pd and Pt are lossy metals that can indeed be expected to damp plasmon resonances^45^.

**Fig. 4:**
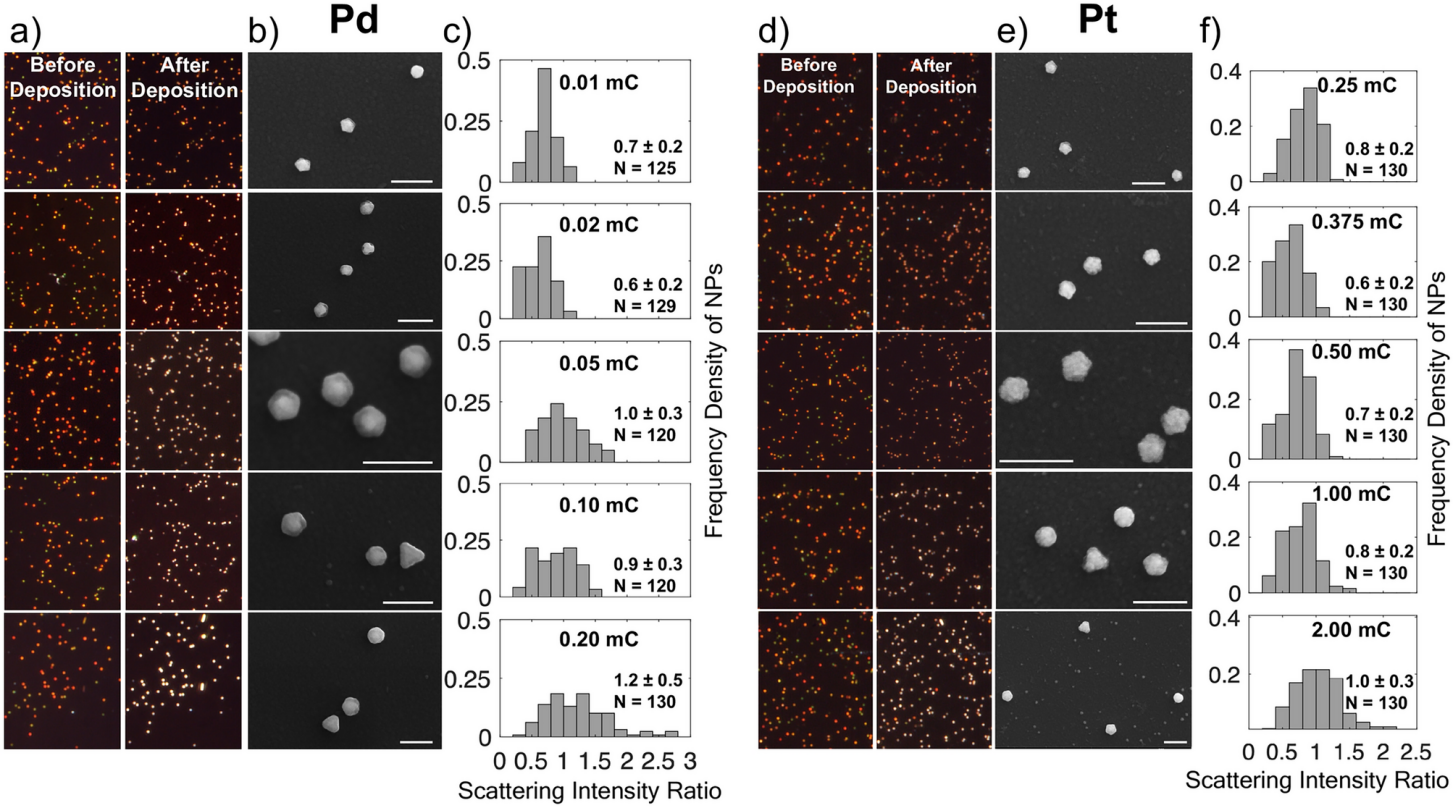
Effect of charge transfer on Pd and Pt electrodeposition on Au NPs, at a constant current of 10 µA and 50 µA, respectively. Dark field optical scattering images from the same region before and after deposition for **a**) Pd and **d**) Pt. SE SEM images of representative NPs after deposition of **b**) Pd and **e**) Pt and LSPR intensity ratio (after deposition divided by initial), with the average, standard deviation, and number of NPs (N) reported on each normalized histogram for **c**) Pd and **f**) Pt. Scale bars, 400 nm.

Systematically changing the charge transfer, thereby the thickness of the metal layer, leads to a systematic change of the bimetallic structure’s optical properties. Specifically, the total charge transfer was varied with a constant current (10 µA for Pd and 50 µA for Pt) and both color images and scattering spectra for > 100 NPs were acquired in air before and after deposition, as shown in Fig. 4. On the color camera, a dimming of the NP color is observed at low charge transfer, followed by a brightening to a white scattering. The trend agrees with the scattering intensity ratio obtained with a spectrometer and CCD, as shown in the histograms of Fig. 4, where the average ratio value initially drops to a minimum of 0.6 for both metals, and then increases up to 1 and 1.2 for Pd and Pt, respectively. The histograms of intensity ratio show a good homogeneity of deposition, but there is more variation in intensity ratio than there is in NP size, and this variation increases as charge transfer increases. At higher charge transfers, islands of Pd and Pt nucleate and grow on the ITO, potentially due to the depletion of ions near the Au NPs; this effect at least partially explains the slowing down of the shell growth visible in Fig. 2.

**Fig. 5:**
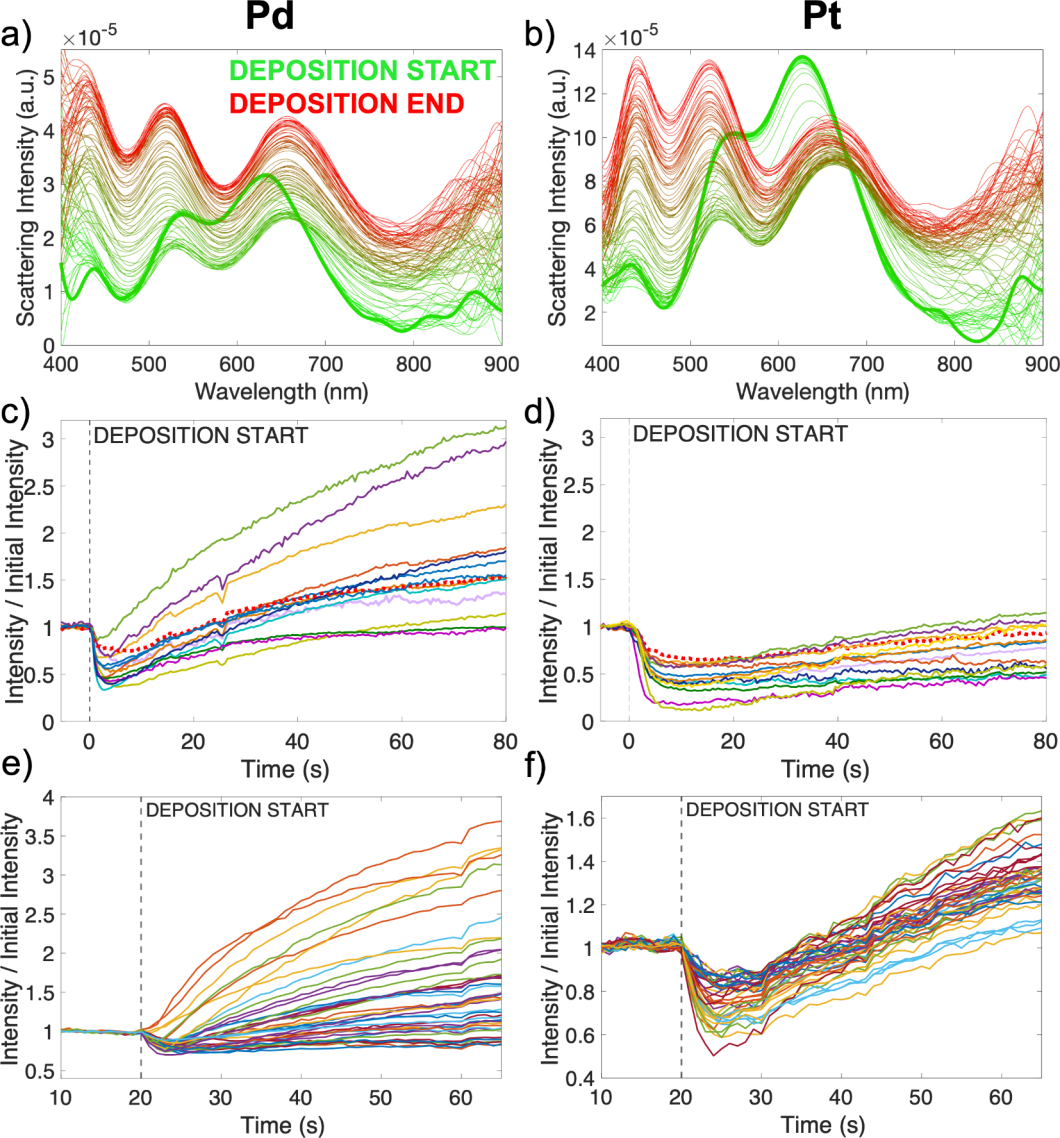
Tracking of NP scattering during electrodeposition. **a**) Scattering spectrum with time of deposition of **a**) Pd and **b**) Pt on a single Au NP; time intervals are 0.5 s from 0 to 80 s. **c**) Scattering intensity divided by initial intensity of the main LSPR peak (acquired with a spectrometer and CCD) against deposition time for **c**) Pd and **d**) Pt on multiple single Au NPs; the dotted traces in red are from the NPs shown in **a**) and **b**). Total scattering intensity (acquired with a color camera) divided by initial intensity against deposition time for **e**) Pd, and **f**) Pt on several single Au NPs.

Alternatively, the scattering of single NPs can be measured during deposition; this approach indicates metal growth and reveals its kinetics. Representative time series for Pd and Pt deposition on single Au NPs are shown in Fig. 5a and b. We report the scattering intensity ratio for both metals as a function of time in Fig. 5c and d. The scattering intensity decreases immediately after the deposition begins, with no waiting period. Experiments conducted in Pt- and Pd-free background electrolyte (Supplementary Fig. S13) show no change when current is switched on or off, demonstrating that the changes in scattering intensity seen here occur due to the deposition of metal on Au, and confirm that charge density tuning effects^11^ are not prominent here. As deposition time increases, the metal shell grows and the scattering intensity increases (Fig. 5), following the trends observed for the fixed charge transfer intervals (Fig. 4).

The deposition can be tracked with a color camera, showing the same trends. The scattering ratio from the sum of RGB channels of a color camera (Fig. 5e and f) tracks the scattering ratio observed for the low energy peak intensity (Fig. 5c and d). However, the absolute values of scattering ratio are higher for the color camera measurements due to increased scattering at low wavelength as the deposition progresses (Fig. 5a and b).

**Fig. 6:**
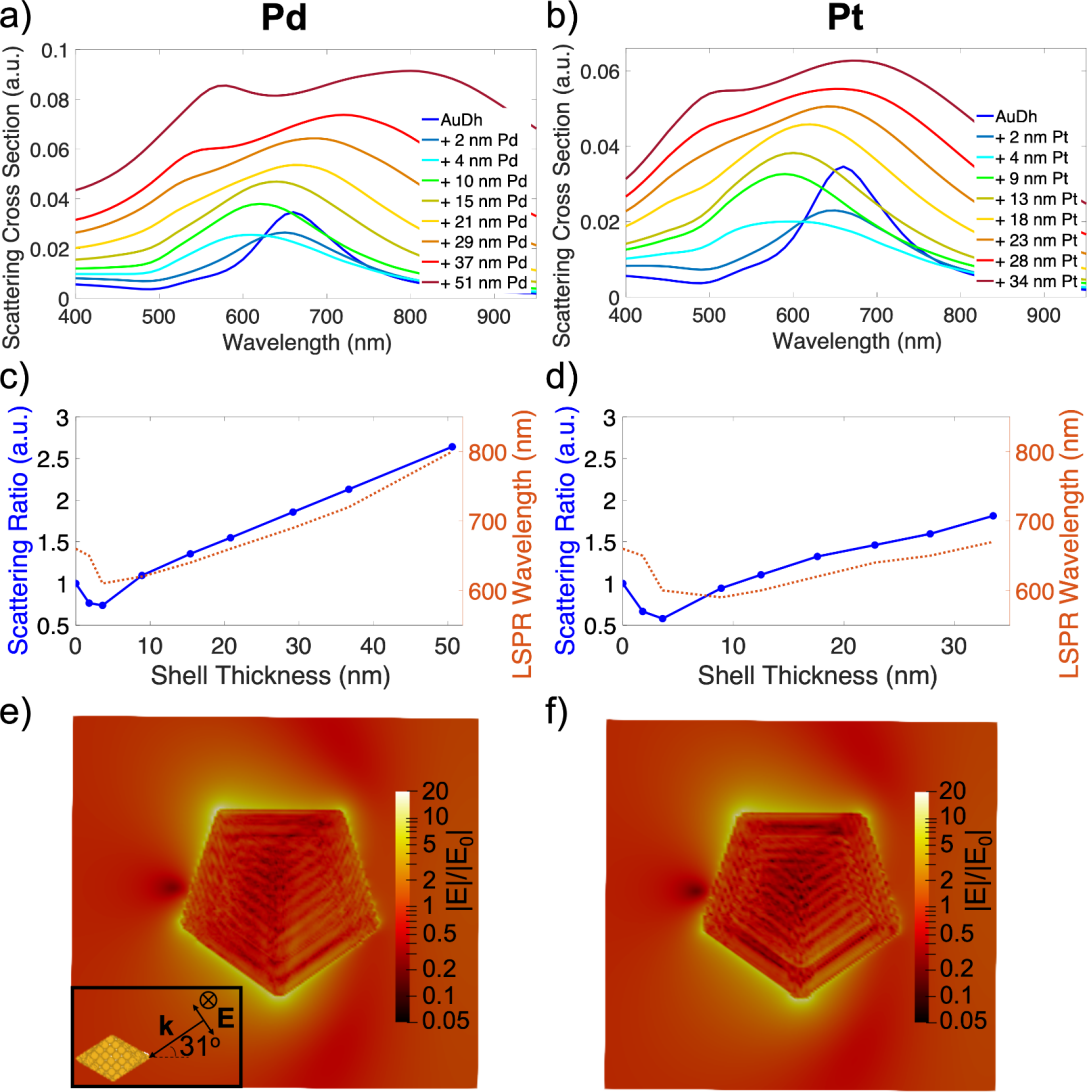
Calculated scattering cross sections for a 136.6 nm decahedral Au NP core with conformal shells of **a**) Pd or **b**) Pt of varying thicknesses corresponding to those reported in Fig. 2. Scattering intensity ratio and LSPR wavelength plotted against shell thickness for **c**) Pd and **d**) Pt on Au NPs. Electric field distribution maps for **e**) Pd (29 nm thickness at a wavelength of 690 nm) and **f**) Pt (23 nm thickness at a wavelength of 640 nm) shells on Au NPs confirming that the highest wavelength peak is the dipolar LSPR.

Numerical simulations support the observed optical changes upon deposition. Scattering cross sections and near-field distributions of conformal shells of either Pd or Pt of varying thicknesses up to 50 nm, matching the experimental range, on a 136.6 nm tip to tip Au decahedron, were obtained numerically in the discrete dipole approximation using DDSCAT^37^ (Fig. 6). This size is consistent with the experimentally observed size when considering the different degree of rounding and the 2 nm interdipole distance. To account for substrate effects, the effective medium refractive index was set to 1.3, approximately the weighted average of the refractive indices of air and the ITO substrate^46^. Near field calculations (Fig. 6e and f) confirm that the lowest energy peak, at ~ 650 nm is, as expected, the azimuthal dipole.

The numerical results demonstrate that the addition of a Pd or Pt shell results in non-monotonic shifts of both the dipole resonance energy and intensity (Fig. 6a and b). We emphasize this trend in Fig. 6c and d by plotting the dipole scattering intensity ratio and resonance wavelength as a function of shell thickness for both deposited metals. The scattering intensity ratio is defined as it was experimentally, *i.e.*, as the scattering intensity at the dipole resonance maximum at a given shell thickness, divided by the scattering intensity of the main LSPR peak for a bare Au NP. A blueshift and decrease in the scattering intensity ratio occurs up to a thickness of ~ 5 nm, followed by a monotonic redshift and increase in the intensity ratio. In the case of Pd, the addition of a shell of Pd leads to higher cross sections and longer resonance wavelengths than for Pt. Mie theory calculations for spherical NPs also support these trends in scattering ratio and LSPR shift as shell thickness increases (Supplementary Fig. S14).

These numerical results match well with experiments, save a few discrepancies. Indeed, Fig. 4 shows a decrease followed by an increase in scattering intensity, as predicted numerically. However, the absolute value of the intensity ratio is smaller experimentally than numerically. Next, the tracking results of Fig. 5 also match the predictions of decreased then increased intensity, similarly with a lower intensity than in numerical simulations. Also, single particle spectral tracking reveals the expected redshift, although without the predicted initial blueshift.

Both discrepancies are minor and likely due to differences between simulations and experiments, specifically the refractive index surrounding the NPs and the lumpy character of the Pd or Pt shell. Numerical results for particles in a homogeneous refractive index environment of n = 1 (Supplementary Fig. S15) show a greater scattering ratio for a given shell thickness, as well as a greater blue shift (and smaller eventual red shift) compared to particles in n = 1.3 (chosen to approximate the substrate effects, Fig. 6). Similarly, systematically lowering the refractive index in a simpler simulation approach (Supplementary Fig. S14) results in a greater scattering ratio for a given thickness, and a greater initial blue shift (and smaller eventual red shift). Refractive index choice in simulations, in addition to its distribution around the NP, may thus explain the differences in scattering ratio and trends in peak shift observed. There are also likely contributions from the shell smoothness in numerics and lumpiness in experiments. Our previous work on Cu on Au NPs showed that the growth of smooth shells resulted in a smaller red shift than for more pointed, multi lobe decorated structures of a similar size^24^. The shells of Pd and Pt initially growing in a lumpy manner (as seen in Fig. 2) could explain why the numerically predicted initial blue shifts are not observed experimentally.

Lastly, electrodeposited Pd and Pt on Au NPs are stable in air and in the electrolyte solution after synthesis, a promising start for their potential applications. In air, no differences were observed in the Pd or Pt on Au NP scattering spectra or color camera images between 0 and 24 h after deposition (Supplementary Fig. S16). Additionally, samples were held in the electrolyte solution post deposition and the scattering intensity of several single particles were measured from the color camera intensity measurements; Pd scattering spectra and intensity remained stable, while only negligible changes were seen for Pt (Supplementary Fig. S17).

## Conclusion

We achieved Pd and Pt shells on Au NPs with controllable size and optical properties using fixed current electrodeposition. A good deposition homogeneity was obtained through the optimization of multiple deposition parameters. Tuning the charge transfer led to controllable shell thicknesses with narrow size distributions. The deposition of thin shells initially leads to a damping of the Au LSPR and a decrease in scattering intensity, followed by an increase in scattering intensity and spectral red shift as the shell grows larger. These changes in plasmonic behavior were compared to numerically simulated trends confirming that they are caused by the growth of Pd or Pt on the surface of Au NPs. Both Pd and Pt on Au NPs were found to be stable after deposition, in air and in solution. Overall, these results highlight the advantages of electrodeposition to produce bimetallic plasmonic NPs and open the door for the future synthesis and investigation of photocatalytic materials.

## Supplementary Information

Supplementary Information.

## Data Availability

The datasets generated during and/or analysed during the current study are available from the corresponding author on reasonable request.
