# Supplementary material for: Controllable synthesis of Pd and Pt shells on Au nanoparticles with electrodeposition
Source: Sci Rep. 2025 Jan 8;15:1292. doi: 10.1038/s41598-024-84476-z (PMC11711627; doi:10.1038/s41598-024-84476-z)
Supplement: Supplementary file 1 — Supplementary Information. [file 41598_2024_84476_MOESM1_ESM.pdf]

# Supplementary Information for

# Controllable Synthesis of Pd and Pt Shells on Au Nanoparticles with Electrodeposition


Mohsen Elabbadi,[1,2] Christina Boukouvala,[1,2] Emilie Ringe[1,2]*

1. Department of Materials Science and Metallurgy, University of Cambridge, 27 Charles Babbage Road, Cambridge, United Kingdom, CB3 0FS
2. Department of Earth Sciences, University of Cambridge, Downing Street, Cambridge, United Kingdom, CB2 3EQ

*Optimization of Deposition Conditions*

The electrodeposition setup shown in Supplementary Figure 1a and b allowed, separately, the control of the substrate NP, electrolyte concentration, and deposition current.

The application of a current (10 μA for 5 s as used in a typical deposition of Pd) in the background electrolyte in the absence of $Na_2PdCl_4$ or $Na_2PtCl_4$ resulted in no change in the dark field color camera response or any visible shell on Au NP in SEM images. When $Na_2PdCl_4$ or $Na_2PtCl_4$ were introduced, shells were seen covering the Au NP in SEM images and changes in color camera response were observed, confirming that these changes arise from the deposition of Pd or Pt (main paper, Figure 3). Compared to the standard experiments performed under illumination, the absence of illumination during deposition (Supplementary Figures S2 and S3) resulted in no difference in the color camera images or SEM morphology, suggesting there is no impact of photodeposition.

The treatment of the Au NPs with $O_2$/Ar plasma (5 min at 20 W) had a profound impact on deposition. Without this treatment, negligible changes in optical response or in SEM images were found, as shown in Supplementary Figures S4 and S5; indeed, all the preliminary investigations of deposition on untreated NPs failed to achieve deposition localized on Au NP and not just all over the ITO substrate.

Selecting the lowest concentration (0.1 mM) of $Na_2PdCl_4$ and $Na_2PtCl_4$ solution resulted in very little deposition on of Pd or Pt on Au, as shown in Supplementary Figures S6 and S7. Using the qualitative dark field color camera images, only a small change in optical properties was seen after deposition compared to the middle concentrations used, and little deposition was seen in SEM images, suggesting the depletion of metal ions at the electrode (supported by the chronopotentiometry traces in Supplementary Figure S12b and d).

High concentrations (2.0 mM) were around the solubility limit for both metal salts and led to no difference (versus 1.0 mM) in Pt and a minimal increase in Pd deposition (versus 0.5 mM). Concentrations of 0.5 mM for $Na_2PdCl_4$ and 1.0 mM for $Na_2PdCl_4$ were used in all subsequent experiments thus minimizing amount of precursor used without compromising the deposition.

The deposition current was varied by an order of magnitude higher and lower while maintaining a constant charge transfer: 0.05 mC for Pd and 0.50 mC for Pt; results are shown in Supplementary Figures S8 and S9. The chronopotentiometry traces of Pd and Pt deposition in Supplementary Figure S12a and c show a decrease in deposition potential as (reduction) current is lowered. At lower reduction current currents, a greater change in the color camera images was seen and more metal deposited in SEM, accompanied by increased nucleation of Pd on the ITO substrate. In controlled current electrodeposition, a decrease in current is compensated for by an increase in deposition potential. This changes the deposition overpotential which directly impacts nucleation density and homogeneity in island electrodeposition.[1,2] A higher (more positive) overpotential results in a lower nucleation driving force and more progressive nucleation across time, leading to more heterogeneity in deposition and nucleation on the substrate (as in Supplementary Figure S8). In contrast, deposition with a high current resulted in a visibly amount of metal deposited. A more negative overpotential is expected to result in an increase in nucleation density, favoring nucleation over growth of existing nuclei.[1,3] It is possible that for such short times, there is depletion of metal ions at the surface of Au NPs, additionally hindering the growth of the bimetallic NPs. The intermediate currents were thus selected, with the added benefit that they allowed deposition times (5 - 50 s) compatible with *in situ* measurements of the optical response.

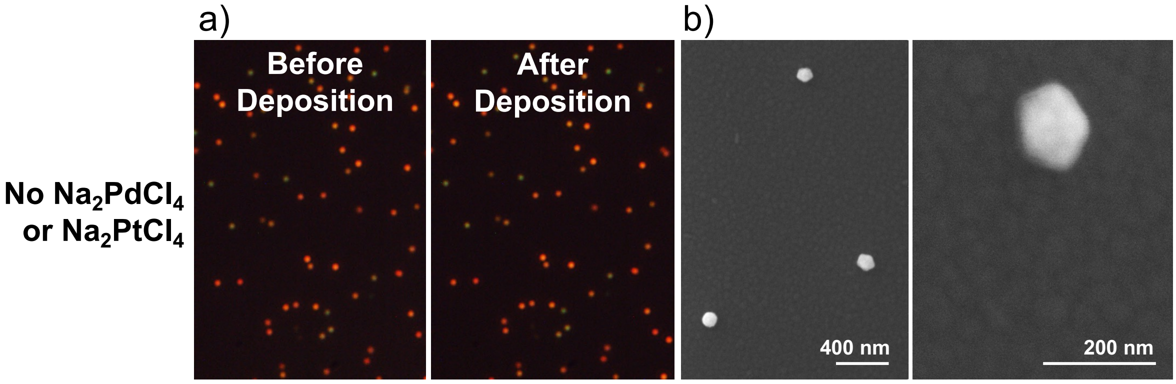


**Supplementary Figure S1.** Effect of applying a current in the absence of $Na_2PdCl_4$ or $Na_2PtCl_4$ on electrodeposition on Au NPs, at a constant current of 10 µA for 5 s. a) Dark field optical scattering images from the same region before and after deposition, and b) SE SEM images of representative NPs after deposition.

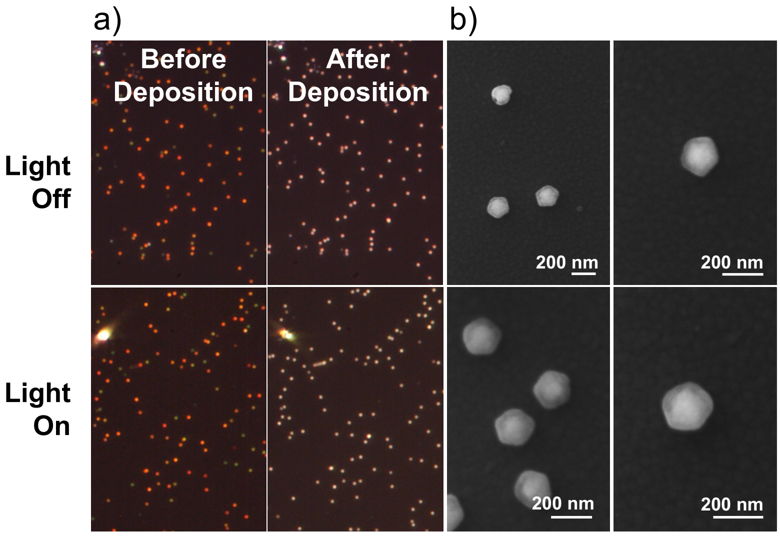


**Supplementary Figure S2.** Effect of illumination on Pd electrodeposition on Au NPs, at a constant current of 10 µA for 5 s. a) Dark field optical scattering images from the same region before and after deposition, and b) SE SEM images of representative NPs after deposition.

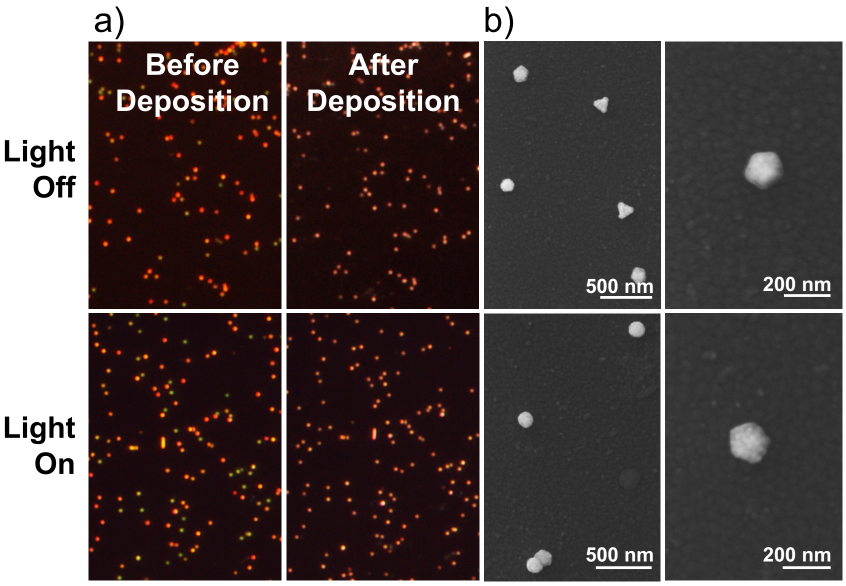


**Supplementary Figure S3.** Effect of illumination on Pt electrodeposition on Au NPs, at a constant current of 50 µA for 10 s. a) Dark field optical scattering images from the same region before and after deposition, and b) SE SEM images of representative NPs after deposition.

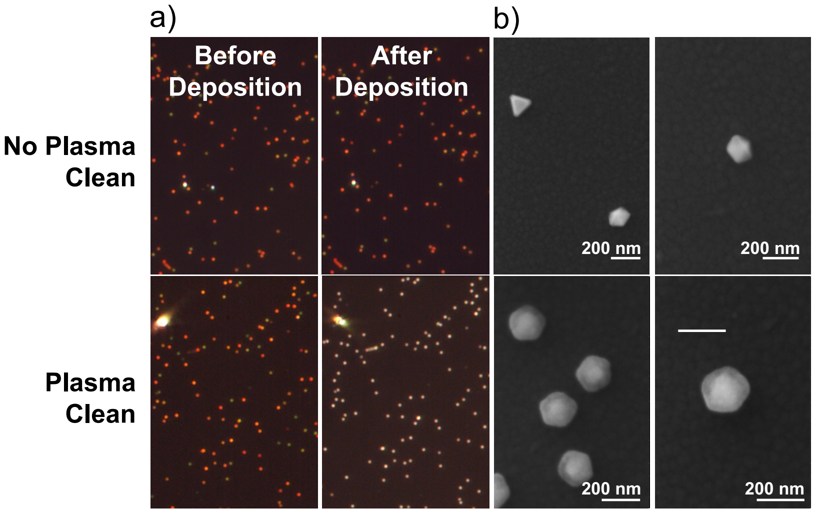


**Supplementary Figure S4.** Effect of (5 mins, 20 W Ar/$O_2$) plasma treatment on Pd electrodeposition on Au NPs, at a constant current of 10 µA for 5 s. a) Dark field optical scattering images from the same region before and after deposition, and b) SE SEM images of representative NPs after deposition.

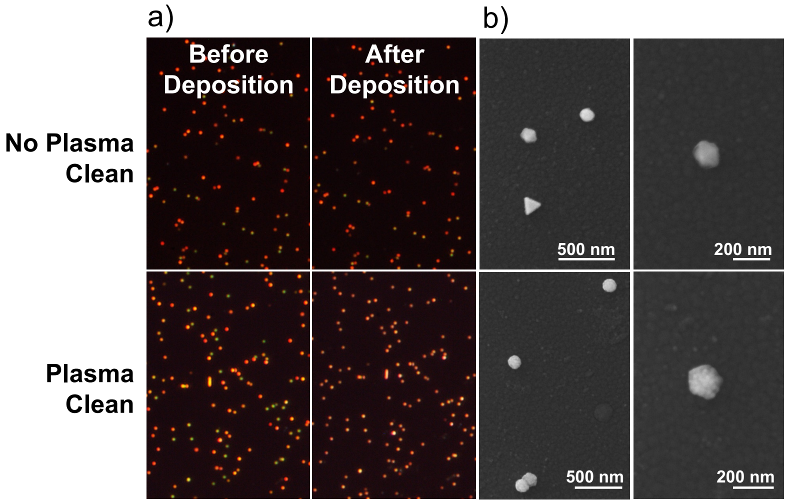


**Supplementary Figure S5.** Effect of (5 mins, 20 W Ar/$O_2$) plasma treatment on Pt electrodeposition on Au NPs, at a constant current of 50 μA for 10 s. a) Dark field optical scattering images from the same region before and after deposition, and b) SE SEM images of representative NPs after deposition.

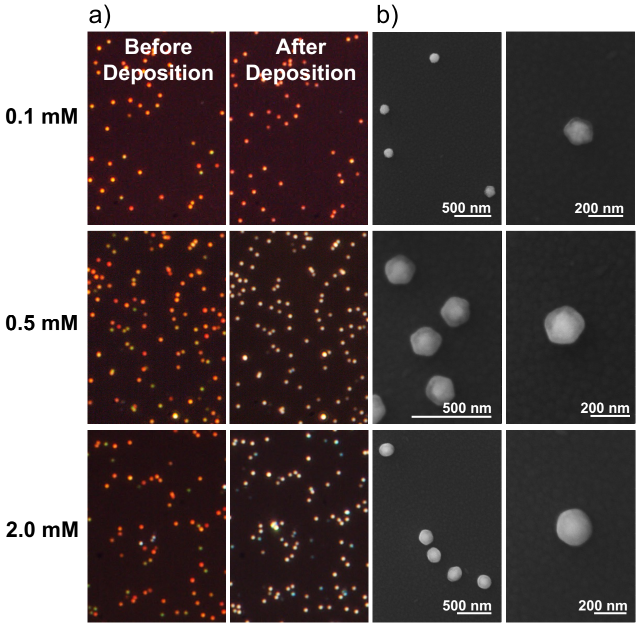


**Supplementary Figure S6.** Effect of $Na_2PdCl_4$ concentration on Pd electrodeposition on Au NPs, at a constant current of 10 μA for 5 s. a) Dark field optical scattering images from the same region before and after deposition, and b) SE SEM images of representative NPs after deposition.

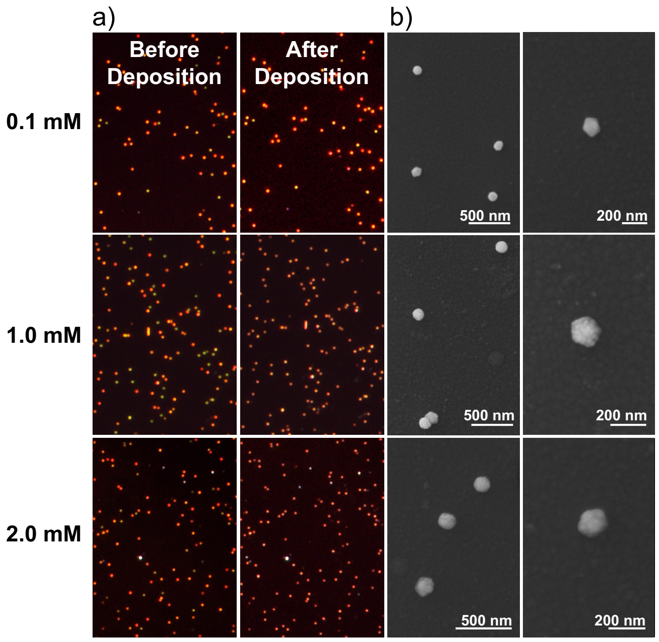


**Supplementary Figure S7.** Effect of $Na_2PtCl_4$ concentration on Pt electrodeposition on Au NPs, at a constant current of 50 μA for 10 s. a) Dark field optical scattering images from the same region before and after deposition, and b) SE SEM images of representative NPs after deposition.

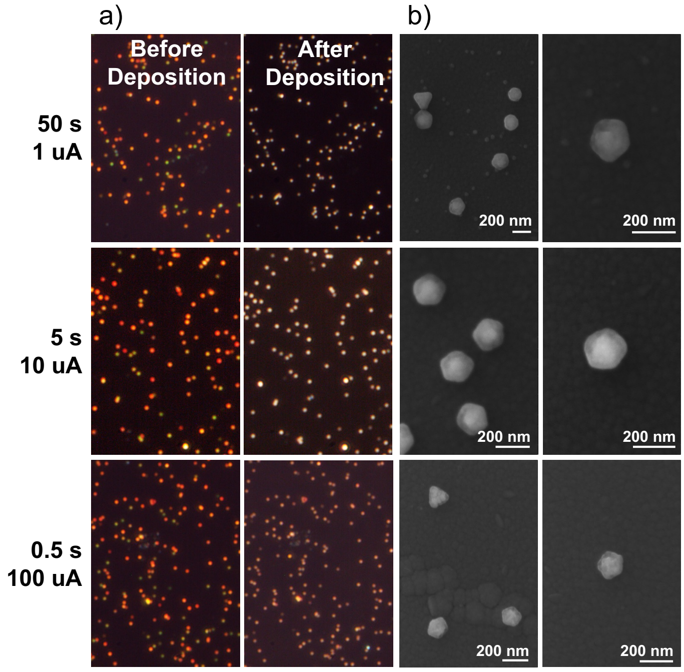


**Supplementary Figure S8.** Effect of current on Pd electrodeposition on Au NPs, at a constant total charge transfer of 0.05 mC. a) Dark field optical scattering images from the

same region before and after deposition, and b) SE SEM images of representative NPs after deposition.

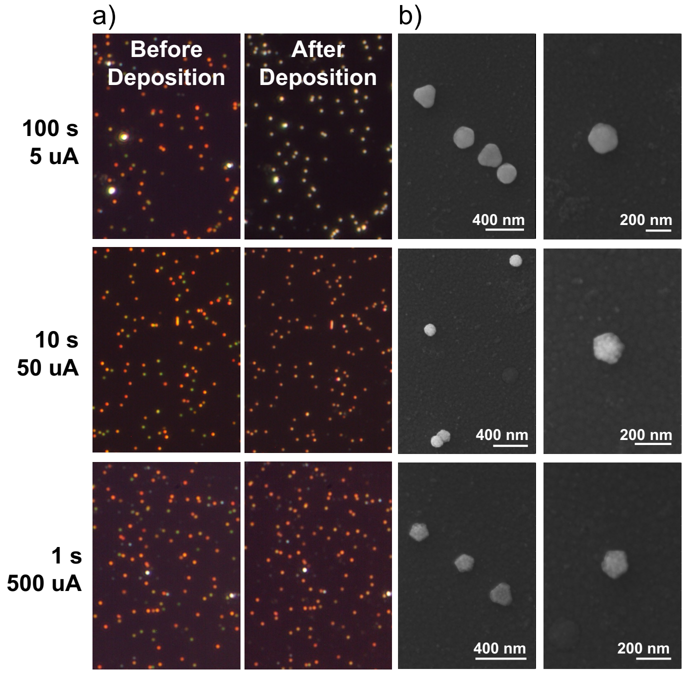


**Supplementary Figure S9.** Effect of current on Pt electrodeposition on Au NPs, at a constant total charge transfer of 0.50 mC. a) Dark field optical scattering images from the same region before and after deposition, and b) SE SEM images of representative NPs after deposition.

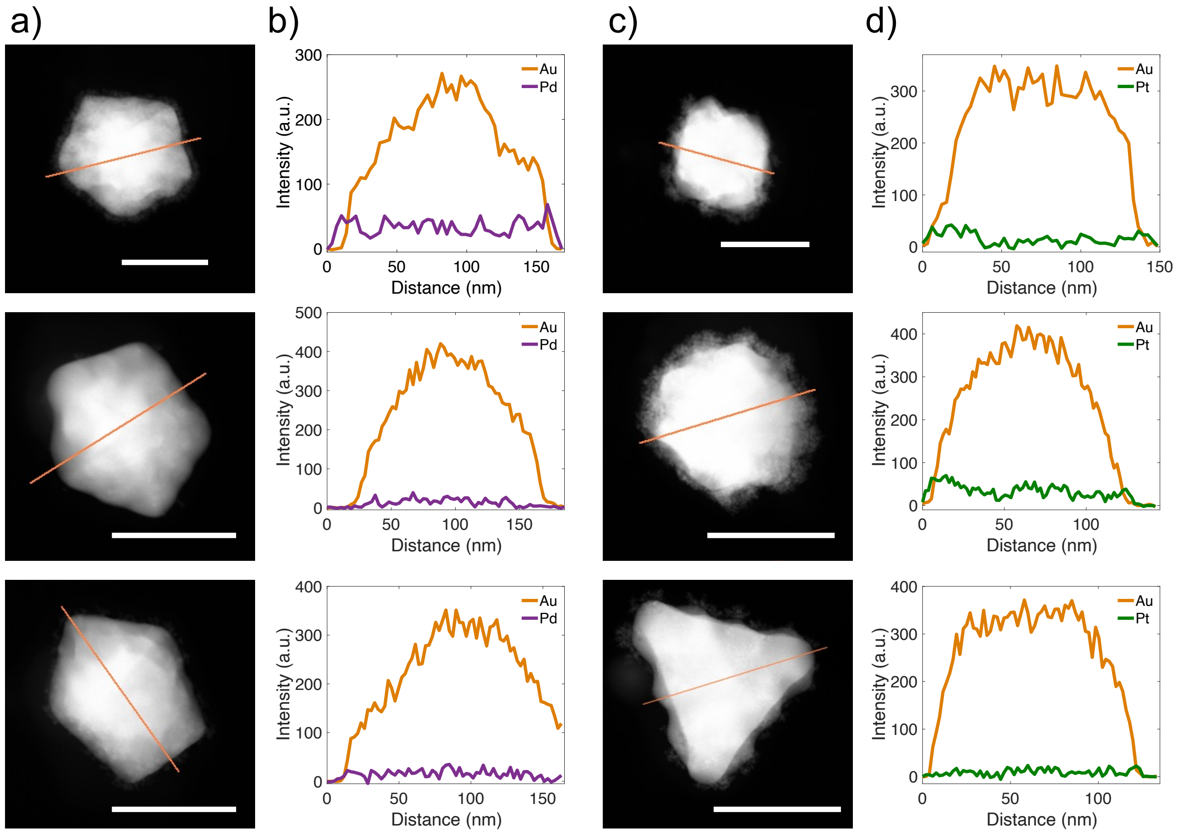


**Supplementary Figure S10.** High angle annular dark field scanning transmission electron microscopy (HAADF-STEM) images (a) and c)) and STEM energy dispersive X-ray spectroscopy (STEM-EDS) line scans (b) and d)) for different NPs. Pd (a) and b)) was deposited for 5 s at 10 µA, while Pt (c) and d)) was deposited for 10 s at 50 µA. Scale bars, 100 nm.

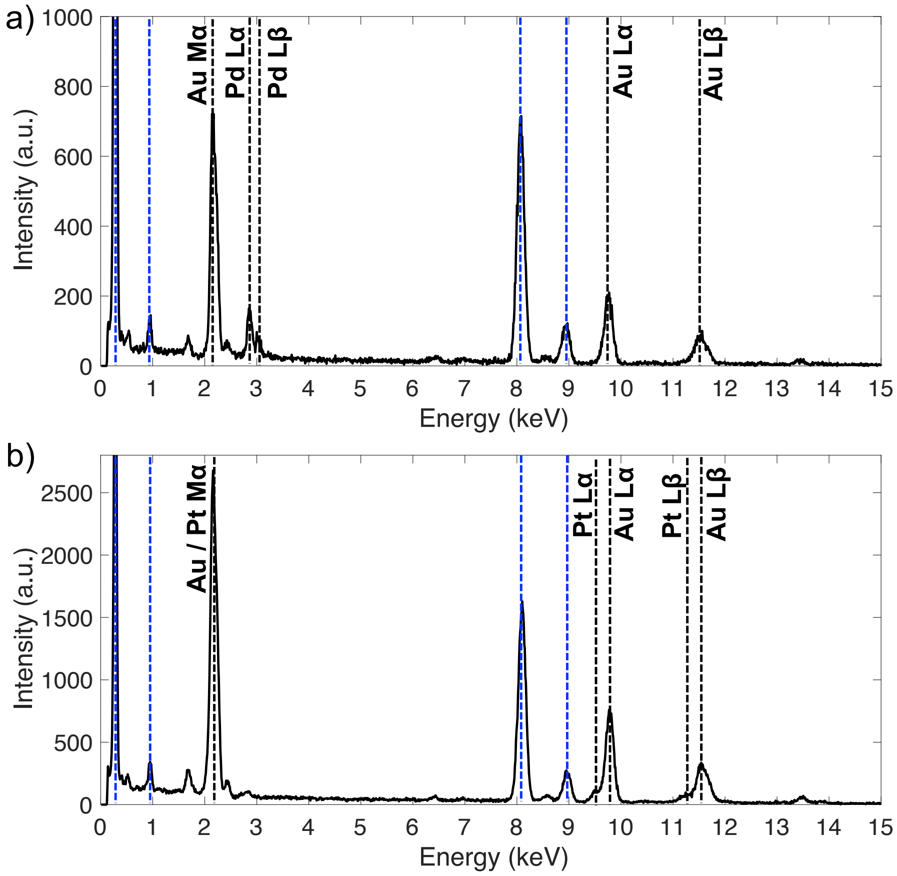


**Supplementary Figure S11.** STEM EDS spectra for a) Pd on Au NP, b) Pt on Au NP deposited on C-coated Cu mesh grids. Blue lines indicate peaks from C or Cu present in the support grid.

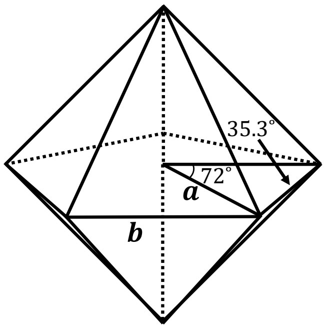


**Supplementary Schematic S1.** Schematic of the perfect decahedron used to model the decahedral Au NPs in main paper, Figure 2, showing the relevant angles.

The volume of a pentagonal bipyramid (decahedron) is given by

$$V_{deca} = \gamma a^3 \qquad (1)$$

where $a$ is the radial corner to center distance and $\gamma$ is given by

$$\gamma = \frac{10sin36^{\circ}cos^2 36^{\circ}tan35.3^{\circ}}{3} \approx 0.908 \qquad (2)$$

The volume of a uniform decahedral shell is thus

$$V_{shell} = \gamma a^3 - \gamma a'^3 \qquad (3)$$

where $a$ is the size of the NP with shell and $a'$ is the size of the core (from simple geometry, $a'$ would be 72 nm for a 130 nm tip to tip NP). This gives a final shell thickness (along the $a$ direction) of

$$t = \sqrt[3]{\frac{V_{shell}+\gamma a'^3}{\gamma}} - a' \qquad (4)$$

Expressing this volume as a function of the edge length $b$, where $a = \frac{1}{2tan30^{\circ}} b$,

$$V = \frac{10sin36^{\circ}cos^2 36^{\circ}tan35.3^{\circ}}{3} \times (\frac{1}{2tan30^{\circ}} b)^3 \approx 0.590\, b^3 \qquad (5)$$

Alternatively, for simplicity one could assume that the volume is that of five regular tetrahedra with edge length $b$ such that

$$V = 5\ \frac{b^3}{6\sqrt{2}} \approx 0.589\ b^3$$

Of course, this assumption of 5 regular tetrahedra leads to a slightly smaller volume given the tetrahedral angle is ~70.5° (not 72 °) and five tetrahedra do not fill space.

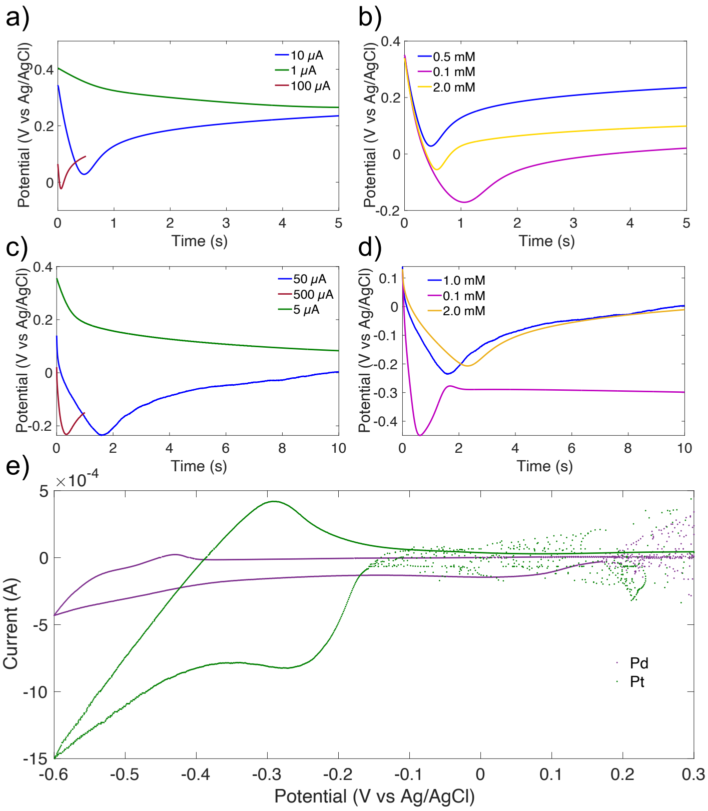


**Supplementary Figure S12.** Chronopotentiometry and cyclic voltammetry curves for Pd and Pt deposition on Au NPs. a) Chronopotentiometry curves for Pd deposition at different currents (fixed total charge transfer of 0.05 mC), b) chronopotentiometry curves for Pd deposition at different concentrations (10 μA, 5 s), c) chronopotentiometry curves for Pt deposition at different currents (fixed total charge transfer of 0.50 mC), d) chronopotentiometry curves for Pt deposition at different concentrations (50 μA, 10 s), e) cyclic voltammograms for Pd and Pt (scan rate 25 mV $s^{-1}$).

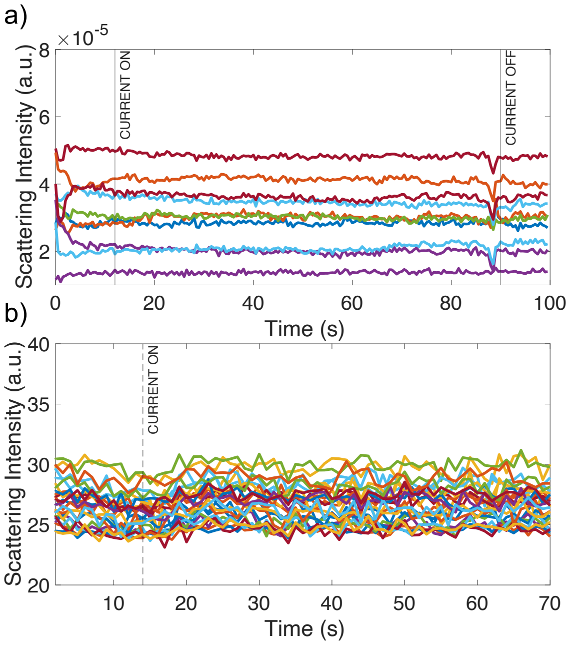


**Supplementary Figure S13.** Scattering intensity of a) LSPR peak of 10 NPs and b) colour camera traces of 25 NPs during a current of 10 μA in the absence of Pd or Pt ions (1 mM $H_2SO_4$).

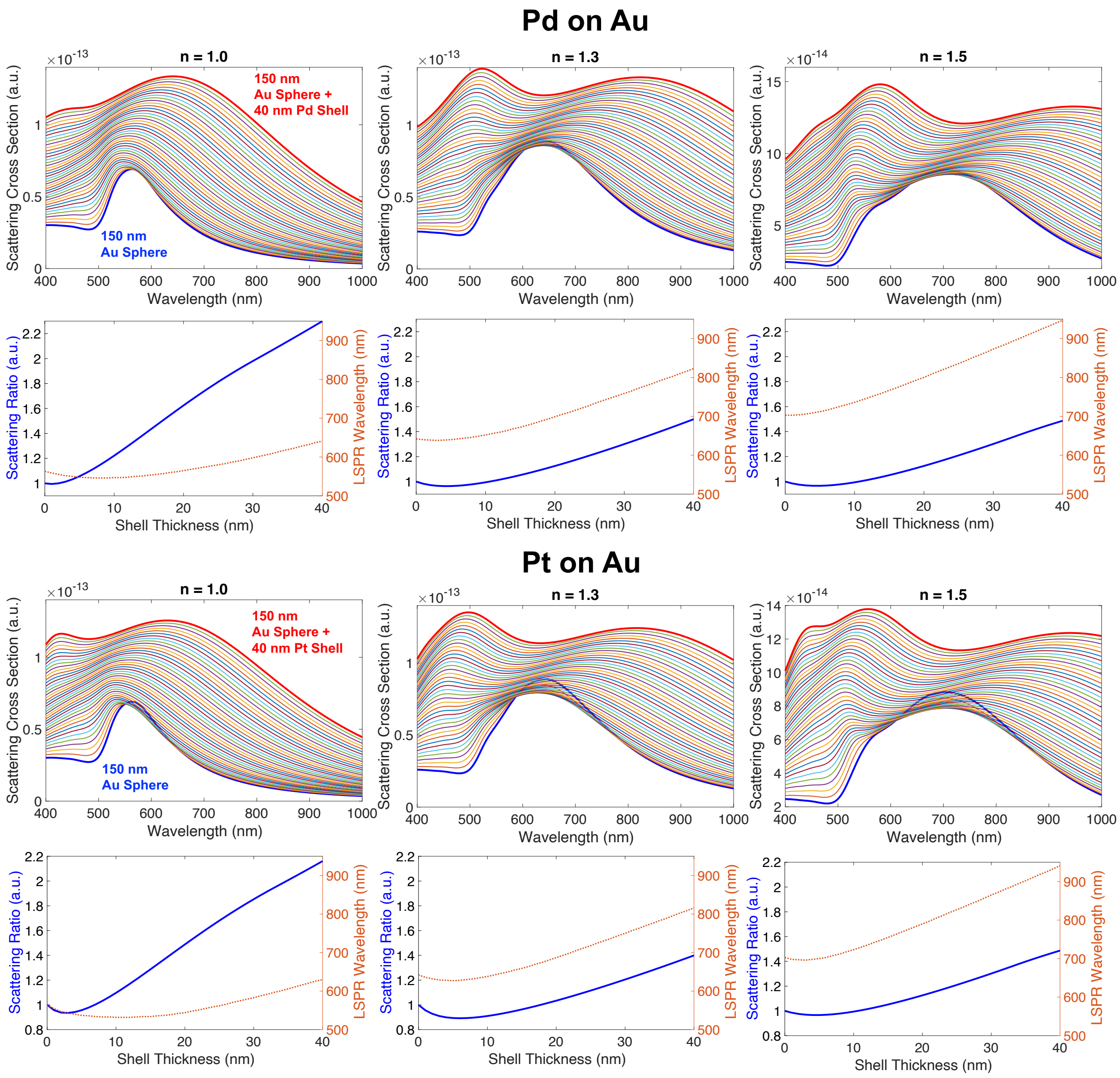


**Supplementary Figure S14.** Mie Theory calculated scattering cross sections and intensity ratios for different Pd or Pt shell thicknesses (intervals are 1 nm from 0 to 40 nm) on a 150 nm diameter Au spherical core at different medium refractive index (n) using STRATIFY.

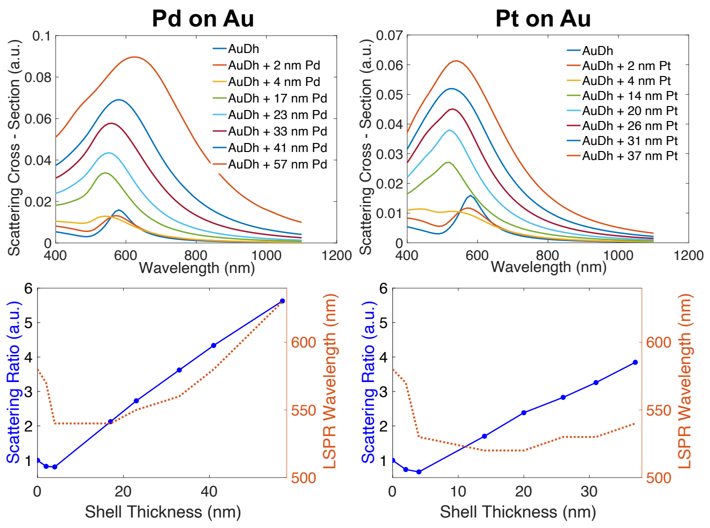


**Supplementary Figure S15.** Scattering cross sections, scattering ratios and LSPR wavelengths for different thicknesses of conformal Pd or Pt shells on 136.6 nm (tip to tip) Au decahedral cores calculated using DDSCAT for a medium refractive index (n) of 1 (results for n=1.3 are presented in the main paper, Figure 6).

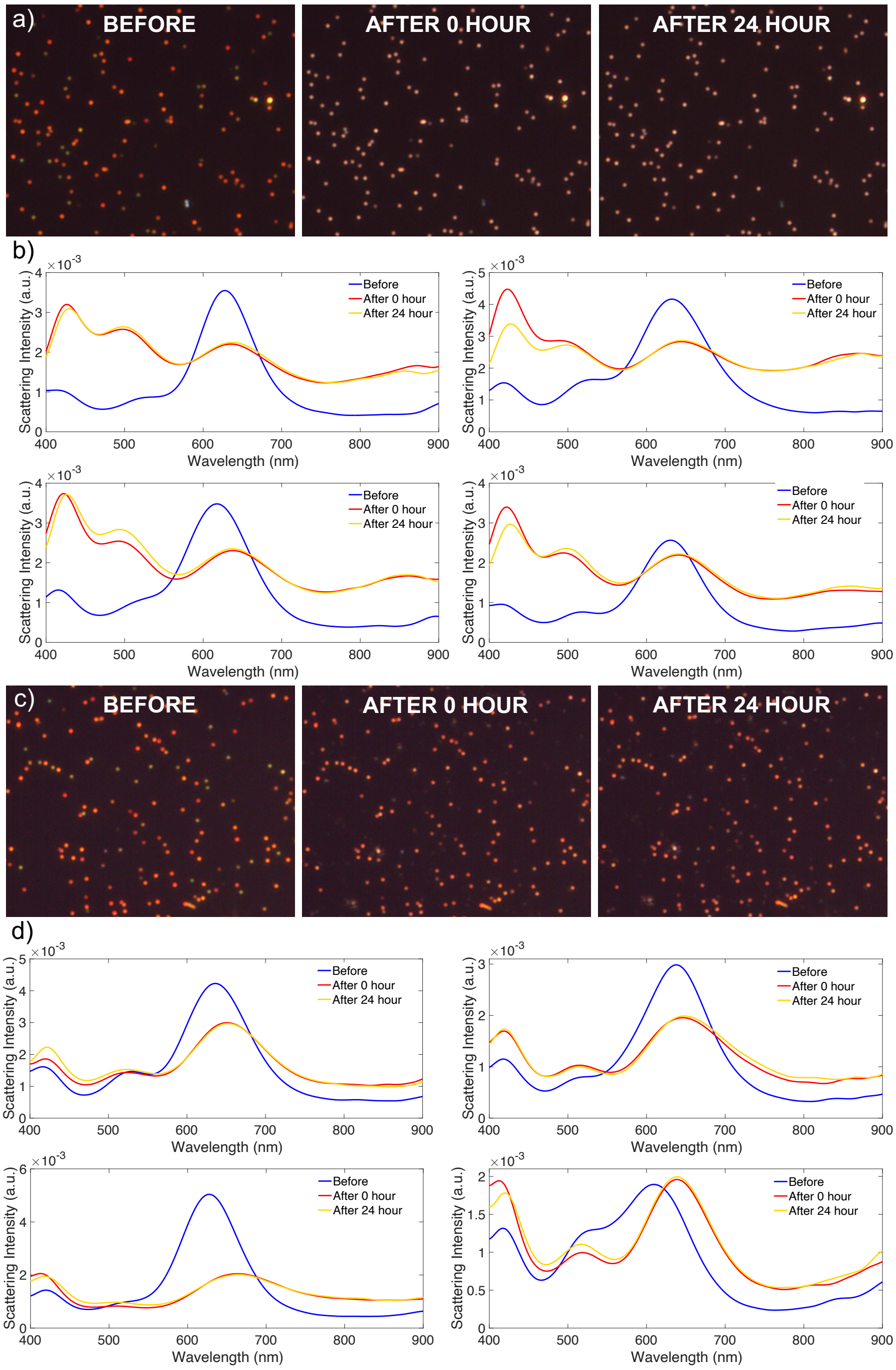


**Supplementary Figure S16.** Stability of Pd or Pt on Au NPs in air after deposition. a) Dark field optical scattering images and b) representative scattering spectra of four Pd on Au NPs before and after 0 and 24 hours post deposition, c) dark field optical scattering images and d) representative scattering spectra of four Pt on Au NPs before and after 0 and 24 hours post deposition.

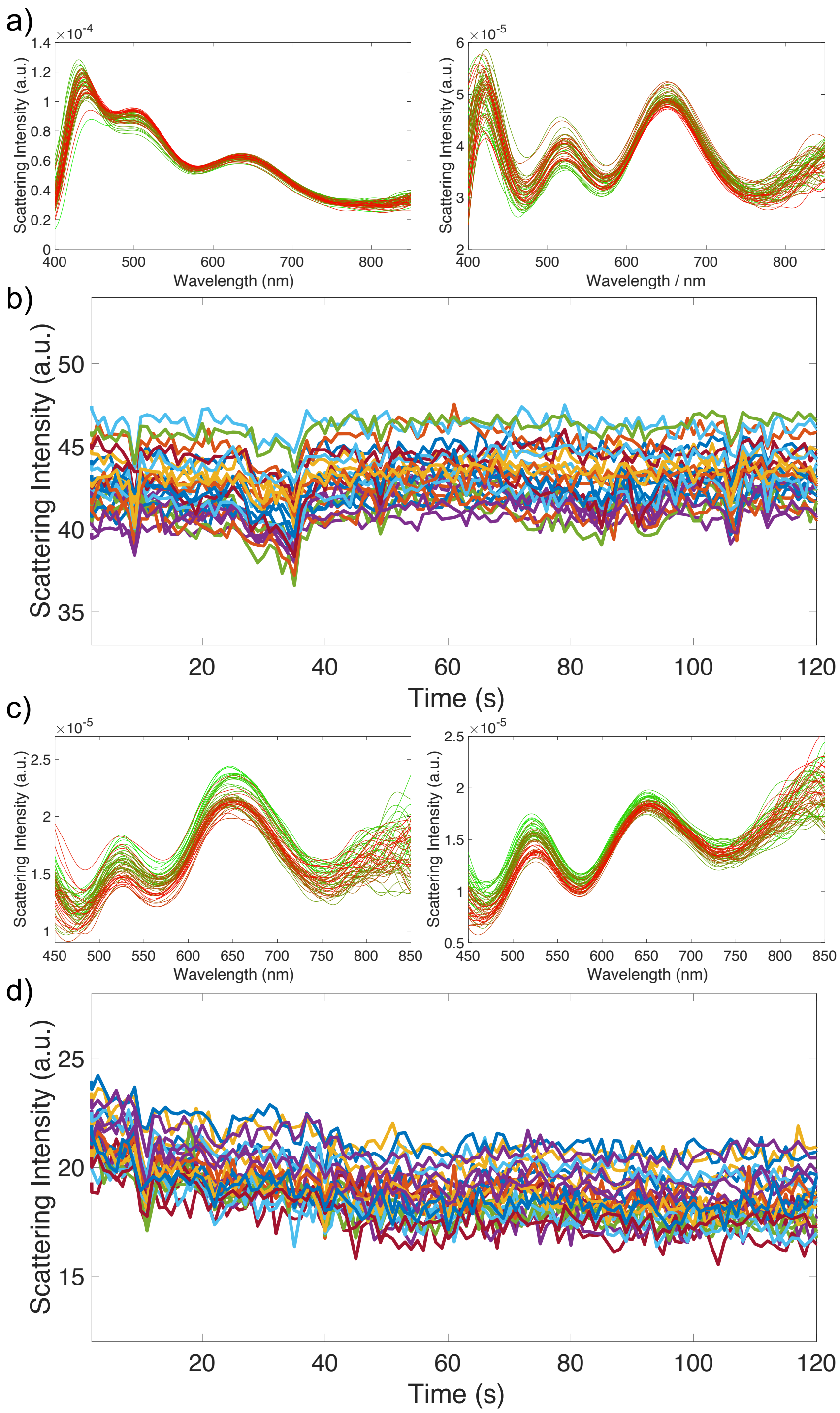


**Supplementary Figure S17.** Stability of Pd or Pt on Au NPs in solution after deposition. a) representative scattering spectra of two Pd on Au NPs and b) colour camera scattering traces of 25 Pt on Au NPs up to 120 s post deposition, c) representative scattering spectra of two Pt on Au NPs and d) colour camera scattering traces of Pt on Au 25 NPs up to 120 s post deposition.

**References**

(1) Guo, L.; Searson, P. C. On the Influence of the Nucleation Overpotential on Island Growth in Electrodeposition. *Electrochim. Acta* **2010**, *55* (13), 4086–4091. https://doi.org/https://doi.org/10.1016/j.electacta.2010.02.038.

(2) Guo, L.; Oskam, G.; Radisic, A.; Hoffmann, P. M.; Searson, P. C. Island Growth in Electrodeposition. *J. Phys. D: Appl. Phys.* **2011**, *44* (44), 443001. https://doi.org/10.1088/0022-3727/44/44/443001.

(3) Elabbadi, M.; Boukouvala, C.; Hopper, E. R.; Asselin, J.; Ringe, E. Synthesis of Controllable Cu Shells on Au Nanoparticles with Electrodeposition: A Systematic in Situ Single Particle Study. *J. Phys. Chem. C* **2023**, *127* (10), 5044–5053. https://doi.org/10.1021/acs.jpcc.2c08910.
